# Supplementary material for: Unveiling the Bioactive Potential of the Invasive Jellyfish Phyllorhiza punctata Through Integrative Transcriptomic and Proteomic Analyses
Source: Biomolecules. 2025 Aug 4;15(8):1121. doi: 10.3390/biom15081121 (PMC12383608; doi:10.3390/biom15081121)
Supplement: Supplementary file 1 [file biomolecules-15-01121-s001.zip › Suplementary Material/FileS3/PEPFOLD4/XP_065065159_11603/clusters.html]

 PEP-FOLD clusters


Download archive of all models  here   

|  |  |  |
| --- | --- | --- |
| # | file | sOPEP |
| # cluster 1 - Size: 60 |  |  |
|  | XP\_065065159\_11603-model1 | -6.44704 |
|  | XP\_065065159\_11603-model1.1 | -6.44704 |
|  | XP\_065065159\_11603-model1.2 | -6.44704 |
|  | XP\_065065159\_11603-model1.3 | -6.44704 |
|  | XP\_065065159\_11603-model1.4 | -6.44704 |
|  | XP\_065065159\_11603-model1.5 | -6.44704 |
|  | XP\_065065159\_11603-model1.6 | -6.44704 |
|  | XP\_065065159\_11603-model1.7 | -6.44704 |
|  | XP\_065065159\_11603-model1.8 | -6.44704 |
|  | XP\_065065159\_11603-model1.9 | -6.44704 |
|  | XP\_065065159\_11603-model1.10 | -6.44704 |
|  | XP\_065065159\_11603-model1.11 | -6.44704 |
|  | XP\_065065159\_11603-model1.12 | -6.44704 |
|  | XP\_065065159\_11603-model1.13 | -6.44704 |
|  | XP\_065065159\_11603-model1.14 | -6.44704 |
|  | XP\_065065159\_11603-model1.15 | -6.44704 |
|  | XP\_065065159\_11603-model1.16 | -6.44704 |
|  | XP\_065065159\_11603-model1.17 | -6.44704 |
|  | XP\_065065159\_11603-model1.18 | -6.44704 |
|  | XP\_065065159\_11603-model1.19 | -6.44704 |
|  | XP\_065065159\_11603-model1.20 | -6.44704 |
|  | XP\_065065159\_11603-model1.21 | -6.44704 |
|  | XP\_065065159\_11603-model1.22 | -6.44704 |
|  | XP\_065065159\_11603-model1.23 | -6.44704 |
|  | XP\_065065159\_11603-model1.24 | -6.44704 |
|  | XP\_065065159\_11603-model1.25 | -6.44704 |
|  | XP\_065065159\_11603-model1.26 | -6.44704 |
|  | XP\_065065159\_11603-model1.27 | -6.44704 |
|  | XP\_065065159\_11603-model1.28 | -6.44704 |
|  | XP\_065065159\_11603-model1.29 | -6.44704 |
|  | XP\_065065159\_11603-model1.30 | -6.44704 |
|  | XP\_065065159\_11603-model1.31 | -6.44704 |
|  | XP\_065065159\_11603-model1.32 | -6.44704 |
|  | XP\_065065159\_11603-model1.33 | -6.44704 |
|  | XP\_065065159\_11603-model1.34 | -6.44704 |
|  | XP\_065065159\_11603-model1.35 | -6.30837 |
|  | XP\_065065159\_11603-model1.36 | -6.30837 |
|  | XP\_065065159\_11603-model1.37 | -6.30837 |
|  | XP\_065065159\_11603-model1.38 | -6.30837 |
|  | XP\_065065159\_11603-model1.39 | -6.30837 |
|  | XP\_065065159\_11603-model1.40 | -6.30837 |
|  | XP\_065065159\_11603-model1.41 | -6.30837 |
|  | XP\_065065159\_11603-model1.42 | -6.30837 |
|  | XP\_065065159\_11603-model1.43 | -6.30837 |
|  | XP\_065065159\_11603-model1.44 | -6.30837 |
|  | XP\_065065159\_11603-model1.45 | -6.30837 |
|  | XP\_065065159\_11603-model1.46 | -6.30837 |
|  | XP\_065065159\_11603-model1.47 | -6.30837 |
|  | XP\_065065159\_11603-model1.48 | -6.30837 |
|  | XP\_065065159\_11603-model1.49 | -6.30837 |
|  | XP\_065065159\_11603-model1.50 | -6.30837 |
|  | XP\_065065159\_11603-model1.51 | -6.30837 |
|  | XP\_065065159\_11603-model1.52 | -6.30837 |
|  | XP\_065065159\_11603-model1.53 | -6.30837 |
|  | XP\_065065159\_11603-model1.54 | -6.30837 |
|  | XP\_065065159\_11603-model1.55 | -6.30837 |
|  | XP\_065065159\_11603-model1.56 | -6.30837 |
|  | XP\_065065159\_11603-model1.57 | -6.30837 |
|  | XP\_065065159\_11603-model1.58 | -6.30837 |
|  | XP\_065065159\_11603-model1.59 | -6.27263 |
| # cluster 2 - Size: 37 |  |  |
|  | XP\_065065159\_11603-model2 | -6.31828 |
|  | XP\_065065159\_11603-model2.1 | -6.31828 |
|  | XP\_065065159\_11603-model2.2 | -6.31828 |
|  | XP\_065065159\_11603-model2.3 | -6.31828 |
|  | XP\_065065159\_11603-model2.4 | -6.31828 |
|  | XP\_065065159\_11603-model2.5 | -6.31828 |
|  | XP\_065065159\_11603-model2.6 | -6.31828 |
|  | XP\_065065159\_11603-model2.7 | -6.31828 |
|  | XP\_065065159\_11603-model2.8 | -6.31828 |
|  | XP\_065065159\_11603-model2.9 | -6.31828 |
|  | XP\_065065159\_11603-model2.10 | -6.31828 |
|  | XP\_065065159\_11603-model2.11 | -6.31828 |
|  | XP\_065065159\_11603-model2.12 | -6.31828 |
|  | XP\_065065159\_11603-model2.13 | -6.31828 |
|  | XP\_065065159\_11603-model2.14 | -6.31828 |
|  | XP\_065065159\_11603-model2.15 | -6.31828 |
|  | XP\_065065159\_11603-model2.16 | -6.31828 |
|  | XP\_065065159\_11603-model2.17 | -6.31828 |
|  | XP\_065065159\_11603-model2.18 | -6.31828 |
|  | XP\_065065159\_11603-model2.19 | -6.31828 |
|  | XP\_065065159\_11603-model2.20 | -6.31828 |
|  | XP\_065065159\_11603-model2.21 | -6.31828 |
|  | XP\_065065159\_11603-model2.22 | -6.31828 |
|  | XP\_065065159\_11603-model2.23 | -6.31828 |
|  | XP\_065065159\_11603-model2.24 | -6.31828 |
|  | XP\_065065159\_11603-model2.25 | -6.31828 |
|  | XP\_065065159\_11603-model2.26 | -6.31828 |
|  | XP\_065065159\_11603-model2.27 | -6.31828 |
|  | XP\_065065159\_11603-model2.28 | -6.31828 |
|  | XP\_065065159\_11603-model2.29 | -6.31828 |
|  | XP\_065065159\_11603-model2.30 | -6.31828 |
|  | XP\_065065159\_11603-model2.31 | -6.31828 |
|  | XP\_065065159\_11603-model2.32 | -6.31828 |
|  | XP\_065065159\_11603-model2.33 | -6.31828 |
|  | XP\_065065159\_11603-model2.34 | -6.31828 |
|  | XP\_065065159\_11603-model2.35 | -6.31828 |
|  | XP\_065065159\_11603-model2.36 | -6.31828 |
| # cluster 3 - Size: 2 |  |  |
|  | XP\_065065159\_11603-model3 | -6.27438 |
|  | XP\_065065159\_11603-model3.1 | -6.27438 |
| # cluster 4 - Size: 1 |  |  |
|  | XP\_065065159\_11603-model4 | -6.22299 |
|  |  |  |
